# Restoration of Immune Responsiveness to Glioma by Vaccination of Mice with Established Brain Gliomas with a Semi-Allogeneic Vaccine

**DOI:** 10.3390/ijms17091465

**Published:** 2016-09-02

**Authors:** Sebastiano Gattoni-Celli, M. Rita I. Young

**Affiliations:** 1 Research Service (151), Ralph H. Johnson Veterans Affairs Medical Center, 109 Bee Street, Charleston, SC 29401, USA; gattonis@musc.edu; 2 Department of Radiation Oncology, Medical University of South Carolina, 173 Ashley Avenue, Charleston, SC 29425, USA; 3 Department of Otolaryngology—Head and Neck Surgery, Medical University of South Carolina, 173 Ashley Avenue, Charleston, SC 29425, USA

**Keywords:** cytokines, glioblastoma, immune activation, vaccination

## Abstract

Prior studies had shown the clinical efficacy of a semi-allogeneic glioma vaccine in mice with lethal GL261 gliomas. This was confirmed in the present study. As subcutaneous vaccination resulted in protection against tumor in the brain, the present study assessed the impact of this vaccination of mice bearing established GL261 brain gliomas on their cytokine production upon in vitro exposure to tumor-derived products. Mice with established GL261 brain gliomas were vaccinated subcutaneously with H-2^b^ GL261 glioma cells fused with H-2^d^ RAG-neo cells or with a mock vaccine of phosphate-buffered saline. The results of these analyses show that the presence of GL261 tumor-conditioned medium resulted in increased production of Th1, inflammatory and inhibitory cytokines by spleen cells from control mice and from vaccinated glioma-bearing mice. In contrast, spleen cells of tumor-bearing, mock-vaccinated mice produced lower levels of cytokines in the presence of tumor-conditioned media. However, these results also show that there was not a heightened level of cytokine production in the presence of tumor-conditioned medium by spleen cells of vaccinated mice over the production by spleen cells of control mice. Overall, these results show that vaccination slows growth of the GL261 tumors to the point where GL261-vaccinated mice do not show the signs of morbidly or splenic dysfunction exhibited by unvaccinated, late stage glioma-bearing mice.

## 1. Introduction

Glioblastoma multiforme (GBM) is the deadliest of brain tumors and is one of a group of tumors referred to as gliomas. GBMs make up approximately 15% of all primary brain tumors [1]. Classified as a Grade IV (most serious) astrocytoma, GBMs develop from the astrocytes that support nerve cells, primarily in the cerebral hemispheres, but can develop in other parts of the brain, brainstem, or spinal cord. Each year, more than 3000 Americans are diagnosed with GBMs. Sadly, the cure rate for GBMs is grim, with current therapy prolonging survival but not offering a cure.

Standard treatment for GBM is surgery, followed by combined radiation therapy and chemotherapy. GBM’s capacity to invade and infiltrate normal surrounding brain tissue makes complete resection impossible. After surgery, combined chemo-radiation is used to kill residual tumor cells and to delay recurrence. Temozolomide was approved by the Food and Drug Administration (FDA) in 2005 for treatment of adult GBM; subsequently, the FDA approved Avastin (Bevacizumab) for treating GBM. Nevertheless, with standard of care therapy, the median survival of children and adults with GBM is 15 months, and the five-year survival rate is approximately 10% [2]. It has been reported that Veterans with GBM showed a decreased median survival (6.5 months) relative to a national cohort (9.0 months) [3]. While the reasons for this disparity are unknown, this tumor ultimately takes the life of nearly every affected patient, which mandates exploration of additional therapeutic options for GBM.

The powerful immune reactivity leading to transplant rejection can be attributed to the capacity of T-cells to recognize allogeneic major histocompatibility complex (MHC) molecules as intact structures on the surface of foreign cells [4]. This is largely due to the ability of allogeneic stimulation to mobilize up to 10% of all T lymphocytes, compared with the low precursor T-cell frequencies for most common antigens [5]. Simultaneously, each of the lymphocytes activated through direct allorecognition will also recognize a specific antigenic peptide presented in the context of a self-MHC molecule (MHC restriction). Cross-reactivity between alloantigens and self MHC-restricted antigens can be harnessed to target tumor antigens [4]. This increased capacity to induce alloreactivity has been used in several approaches to stimulate anti-tumor reactivity. For example, in a murine model of colon cancer, vaccination with an allogeneic dendritic cell/syngeneic tumor hybrid was clinically more effective than vaccination with a syngeneic dendritic cell/syngeneic tumor hybrid [6]. In a phase I clinical study of patients with gastrointestinal adenocarcinoma, treatment with a semi-allogeneic vaccine that was generated by fusion of autologous cancer cells with an allogeneic cell line resulted in both immunological and clinical responses [7].

The results of studies indicating the effectiveness of semi-allogeneic cancer vaccines in several cancer models suggest that these vaccines could also be tested as an adjuvant treatment for GBM. Using an orthotopic C57BL/6 mouse model of GBM, our prior study showed that subcutaneous injection of an irradiated GL261×RAG semi-allogeneic hybrid vaccine into mice with established GL261 brain cancer induced an effective anti-tumor clinical response [8]. To further our understanding of the mechanisms of action of semi-allogeneic vaccines, the present study investigated cytokine production of spleen cells from (a) control mice (not bearing tumors); (b) glioma-bearing mice that were mock-vaccinated with phosphate-buffered saline; and (c) mice that were glioma-engrafted and vaccinated with the semi-allogeneic hybrid cells. Cytokine production by spleen cells from these mice was measured after they were cultured with control medium (unstimulated) or cultured with glioma-conditioned medium (stimulated).

Our experimental observations suggest that spleen cells from vaccinated mice exhibited an intact capacity to produce a broad spectrum of cytokines in the presence of tumor-derived products (indistinguishable from that observed in spleen cells of control mice). Spleen cells from mock-vaccinated tumor-bearing mice produced significantly lower levels of cytokines in the presence of mediators derived from glioma cells than did spleen cells of vaccinated mice.

## 2. Results

### 2.1. Clinical Impact of Vaccination

Orthotopically implanted GL261 tumor cells in mice is lethal. In the present study, animals were euthanized once they became ill due to tumor burden. For mice that received the mock vaccine, the interval between tumor implantation and the required euthanasia was between 35 and 70 days. This time line was similar to what was previously described in studies to assess survival times of mock-vaccinated versus tumor-vaccinated mice bearing GL261 brain tumors [8]. To compare the immunological status between vaccinated and mock-vaccinated mice, sample mice that received the semi-allogeneic vaccine were euthanized at the time that mock-vaccinated mice required euthanasia. Consistent with our prior studies [8], vaccination of tumor-bearing mice sustained their healthy appearance longer than was seen for the mock-vaccinated mice.

### 2.2. Th1 Cytokine Production

**Figure 1:**
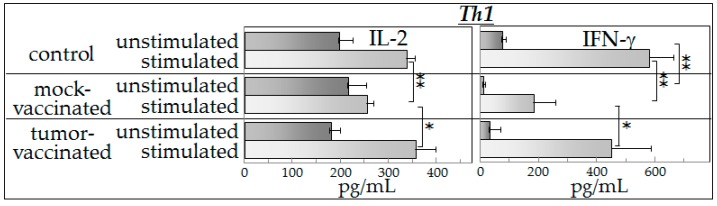
Vaccination of tumor-bearing mice restores production of increased levels of Th1 cytokines in the presence of tumor-derived products. Spleen cells from control mice and from vaccinated or mock-vaccinated glioma-bearing mice were cultured either with control medium (unstimulated) or with glioma-conditioned medium (stimulated). The levels of the Th1 cytokines, IL-2 and IFN-γ, released by the spleen cells into the culture supernatants were then measured. Data shown are means ± SEM, with spleen cell production of cytokines being measured in duplicate for each mouse individually. * *p* < 0.05, ** *p* < 0.01.

Production of cytokines was compared between control mice and tumor-bearing mice that were either mock-vaccinated with phosphate-buffered saline or had received irradiated semi-allogeneic vaccine following the engraftment of GL261 cancer cells in the brain. These cytokine measurements were performed for each mouse individually. Spleen cells from each mouse were cultured in either control medium (unstimulated) or medium containing supernatants from cultured GL261 tumor cells (stimulated). Levels of the Th1 cytokine IL-2 were similar for unstimulated spleen cells from each of the three groups of mice (Figure 1). Comparisons of these IL-2 levels showed medium to large effect sizes (Hedges’ *g*: control vs. sham vaccinated = 0.566; control vs. vaccinated = 0.753; sham vaccinated vs. vaccinated = 1.467). The presence of tumor-conditioned medium increased production of IL-2 by spleen cells from the control mice and from the vaccinated mice almost two-fold, while IL-2 production by the spleen cells from the mock-vaccinated tumor-bearing mice was not significantly affected by the conditioned medium. The results were similar for production of the other Th1 cytokine that was measured, IFN-γ, except that the basal level produced by unstimulated spleen cells from the mock-vaccinated tumor-bearing mice was less than the level produced by spleen cells of control mice. The effect sizes for comparisons of IFN-γ levels were medium to large (Hedges’ *g*: control vs. sham vaccinated = 5.459; control vs. vaccinated = 1.246; sham vaccinated vs. vaccinated = 0.631). Because of the lower basal level of IFN-γ produced by spleen cells of mock-vaccinated tumor-bearing mice, the stimulation index (fold increase) in response to tumor-conditioned medium was greater than for spleen cells from the other groups of mice. Nevertheless, these levels were lower than those measured for spleen cells from either normal control or vaccinated mice.

These results show that glioblastoma-bearing mice have reduced levels of Th1 cytokine production in the presence of tumor-conditioned medium, but the cytokine production in those that were vaccinated is maintained at levels similar to that of control mice. However, these results also show that the increased production of cytokines in the presence of tumor-conditioned medium is not specific to vaccine exposure since the spleen cells from vaccinated mice produced similarly increased levels of Th1 cytokines as did spleen cells of control mice that had not been exposed to either tumor or to the vaccine.

### 2.3. Inflammatory Cytokine Production

**Figure 2:**
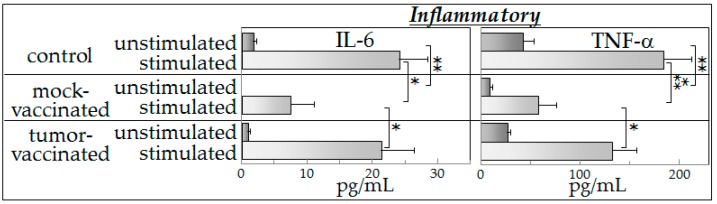
Mock-vaccinated glioma-bearing mice have a diminished capacity to produce inflammatory cytokines in response to the presence of tumor-derived products, but cytokine production is restored in vaccinated tumor-bearing mice. Spleen cells from control mice and from vaccinated or mock-vaccinated glioma-bearing mice were cultured either with control medium or with glioma-conditioned medium and their production of inflammatory cytokines IL-6 and TNF-α was measured. Data shown are means ± SEM, with spleen cell production of cytokines being measured in duplicate for each mouse individually. * *p* < 0.05, ** *p* < 0.01, *** *p* < 0.001.

The pattern of production of the inflammatory cytokines IL-6 and TNF-α (Figure 2) was similar to that seen for the Th1 cytokine IFN-γ (Figure 1). Briefly, spleen cells from the mock-vaccinated tumor-bearing mice produced the lowest basal levels of the inflammatory cytokines (Figure 2). Comparisons of these IL-6 levels showed large effect sizes (Hedges’ *g*: control vs. sham vaccinated = 5.262; control vs. vaccinated = 1.956; sham vaccinated vs. vaccinated = 2.435). The effect sizes for comparisons of TNF-α levels were large (Hedges’ *g*: control vs. sham vaccinated = 4.091; control vs. vaccinated = 2.621; sham vaccinated vs. vaccinated = 5.147). In the presence of tumor-conditioned medium, spleen cells from each of the groups of mice were stimulated to produce increased levels of both of the inflammatory mediators, with the greatest inflammatory cytokine production being by spleen cells of control mice and by spleen cells of vaccinated mice. The least level of production of either IL-6 or TNF-α was by spleen cells from mock-vaccinated tumor-bearing mice. As was seen for the Th1 cytokines (Figure 1), the results in Figure 2 show that tumor vaccination sustains the capacity of spleen cells from tumor-bearing mice to produce increased levels of inflammatory cytokines in the presence of tumor-conditioned medium; these results also show that inflammatory cytokine production by spleen cells of vaccinated mice was not greater than that seen for control mice.

### 2.4. Inhibitory Cytokine Production

**Figure 3:**
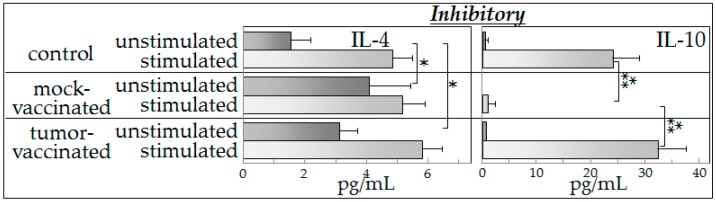
Vaccination of glioma-bearing mice restores an inhibitory cytokine profile that resembles that of control mice and stimulates production of the inhibitory cytokines in response to the presence of tumor-conditioned medium. Spleen cells from control mice and from vaccinated or mock-vaccinated glioma-bearing mice were cultured either with control medium or with glioma-conditioned medium and their production of inhibitory cytokines IL-4 and IL-10 was measured. Data shown are means *±* SEM, with spleen cell production of cytokines being measured in duplicate for each mouse individually. * *p* < 0.05, *** *p* < 0.001.

Compared to results described above for Th1 and inflammatory cytokines, there were differences in patterns of production of the inhibitory cytokines IL-4 and IL-10 by spleen cells of vaccinated and mock-vaccinated mice (Figure 3). Overall, spleen cells produced only low levels of IL-4, regardless of whether the spleen cells were unstimulated or exposed to tumor-conditioned medium, or which group of mice the cells came from. The major difference seen was an increased level of IL-4 production by unstimulated spleen cells of mock-vaccinated tumor-bearing mice compared to unstimulated spleen cells from the other two groups of mice. Comparisons of these IL-4 levels showed large effect sizes (Hedges’ *g*: control vs. sham vaccinated = 2.535; control vs. vaccinated = 2.698; sham vaccinated vs. vaccinated = 1.207). While spleen cells of both control mice and vaccinated mice produced increased levels of IL-4 in the presence of the tumor-conditioned medium, the amount produced was less than that seen for IFN-γ (Figure 1) or the inflammatory mediators IL-6 and TNF-α (Figure 2). Spleen cells from mock-vaccinated mice were not significantly stimulated to produce increased levels of IL-4 when cultured in tumor-conditioned medium. Production of the other inhibitory mediator, IL-10, was low in the absence of stimulation, although measurable levels were produced by control spleen cells and spleen cells of vaccinated mice. The effect sizes for comparisons of IL-10 levels were medium to large (Hedges’ *g*: control vs. sham vaccinated = 1.185; control vs. vaccinated = 0.703; sham vaccinated vs. vaccinated = 1.808). Tumor-conditioned medium stimulated increased IL-10 production by spleen cells of control mice and vaccinated mice, but not by spleen cells from tumor-bearing, mock-vaccinated mice (Figure 3). Similarly to what seen for Th1 and inflammatory cytokines, vaccinated mice resembled the control mice in the capacity of their spleen cells to produce increased levels of inhibitory mediators in the presence of tumor-conditioned medium.

## 3. Discussion

The poor treatment outcome for GBM warrants investigation of alternate treatment approaches. Immunotherapy is one such possible alternative treatment, although this has not been extensively explored for GBM. In our prior studies, a clinical therapeutic response was demonstrated following a subcutaneous (s.c.) inoculation of a semi-allogeneic GL261 glioma vaccine in mice with established GL261 tumors [8]. Analysis of the tumor-inoculation site showed that microglia cells were less numerous within and around the tumor of mock-vaccinated mice, compared to vaccinated mice. In vaccinated mice, conspicuous microglia infiltrates were observed in tumor tissue sections and activated microglia appeared to form a fence along the perimeter of the tumor cells. In contrast, there were no clear differences in T-cell infiltrates between vaccinated and mock-vaccinated mice, although this does not mean that T-cells do not play an important role in the anti-tumor response to vaccination.

The presence of activated microglia at the tumor-inoculation site in vaccinated mice, but the absence of a prominent T-cell infiltrate, prompted studies to assess the peripheral immune impact of tumor presence and vaccination of glioma-bearing mice on their cytokine production in response to tumor-derived products. The rationale for these studies was based on demonstrations of tumor-induced immune dysfunction in a variety of tumors in both humans and in animal models [9,10,11,12]. The present studies were designed to determine if there was immune skewing in glioma-bearing mice and the association between vaccination and changes in the cytokine phenotype. Prior studies have shown direct communications between the immune system and the brain. For example, immune mediators such as IL-1, IL-6, and TNF-α are able to cross the blood-brain barrier [13]. Induction of peripheral inflammation has been shown to trigger neuroinflammation in the CNS [14,15]. Peripheral inflammation results in a concomitant increase in the number of activated microglia and in elevated brain levels of inflammatory mediators IL-1, IL-6 and TNF-α. This study’s assessment of spleen cell cytokine production in mice with brain cancer has as a rationale prior studies showing the capability of monocytes to traffic from the spleen to the brain, thus connecting peripheral immune system to neuronal inflammatory activities [16,17].

The results of the present study showed that the presence of tumor-derived products results in increased cytokine production in either control mice or vaccinated mice. However, spleen cells of tumor-bearing, mock-vaccinated mice produced lower levels of cytokines in the presence of tumor-conditioned medium, consistent with tumor-induced immune suppression. This was most consistently seen for Th1 and inflammatory cytokines, with results of inhibitory cytokines being variable. The reduced cytokine production by spleen cells of tumor-bearing mock-vaccinated mice could not be explained by differences in spleen cell numbers as equal numbers of cells were plated for each of the groups of mice. The present study also showed that both the basal cytokine profile and the stimulated cytokine production by spleen cells of vaccinated mice in the presence of tumor-derived products were similar to those of control mice. While the vaccination restored a normal cytokine phenotype, there was no detectable increase in cytokine production in the presence of tumor-derived products over that seen for normal spleen cells that had not been previously exposed to tumor products. In fact, it seemed ironic that, in the presence of tumor-conditioned medium, spleen cells from control and vaccinated mice not only produced Th1 and inflammatory cytokines, but also produced increased levels of immune inhibitory cytokines. The differences in cytokine production by mock-vaccinated and tumor-vaccinated mice do not represent an across-the-board inability of mock-vaccinated mice to produce cytokines since basal levels of IL-2 that are produced are similar between all of the groups of mice and basal levels of IL-4 that are produced by cells of mock-vaccinated mice are higher than for cells from control mice. In response to stimulation, similar levels of IL-4 are produced by cells from each of the groups of mice. For the purpose of consistency with the clinical analyses that were previously reported with this vaccine model [8], an aliquot of the exact same tumor preparation was used for inoculation into mice in this study as in the prior study. This prior study utilized expression of luciferase by these cells to enable clinical assessment of tumor development in the brain. Since both the mock-vaccinated group and the tumor-vaccinated groups of tumor-bearing mice that were assessed in this study were implanted with this exact same preparation of tumor cells expressing luciferase, the differences in spleen cell cytokine production are not a reflection of luciferase expression. The differences in cytokine production between mock-vaccinated and tumor-vaccinated mice could be due to differences in tumor burden since vaccination slowed growth of the GL261 tumors to the point where GL261-vaccinated mice do not show the signs of morbidly or splenic dysfunction exhibited by unvaccinated, late stage glioma-bearing mice. This could be addressed in subsequent kinetic studies that would allow comparisons of cytokine production across different levels of tumor burden and in the context of histological examination of, not just tumor burden, but also of any immune alterations at the tumor site. Not yet known is what mediators are in the tumor-conditioned media that are responsible for stimulating spleen cell cytokine production. Studies using RNA-Seq are currently ongoing to address possible candidates for this stimulation.

As a multitude of cytokines can cross from the periphery into the brain, studies are needed to test if vaccination can stimulate immune cell release of cytokines that, in turn, lead to neuroinflammation at the tumor site. The most immediate approach to link the effectiveness of peripheral vaccination with reactivity to brain cancer would be to conduct studies measuring circulating cytokine levels in mock-vaccinated and tumor-vaccinated mice. Following determination of circulating cytokine levels, causality between these cytokine phenotypes and any clinical responses would need to be demonstrated through studies such as to neutralize key candidate cytokines with antibody or to use select cytokine knock-out mice.

The in vitro studies described in this report, while correlative, are first steps to better understand the vaccine-driven immunological processes which confer protection against GBM. Continuation of these initial studies is warranted by the recognition that immunotherapy has emerged as an important adjuvant in the therapeutic armamentarium of clinicians against GBM [18], and support the goal of translating our immunotherapy approach into clinical studies.

## 4. Materials and Methods

### 4.1. Culture Medium

Cell culture media consisted of 1× Dulbecco’s modified Eagle’s medium (DMEM, Invitrogen, Grand Island, NY, USA), containing 4.5 g/L d-glucose and l-glutamine, supplemented with 10% fetal bovine serum (FBS, Sigma, St. Louis, MO, USA) and 1× antibiotic antimycotic solution (Sigma) containing penicillin, streptomycin and amphotericin B.

### 4.2. Cells

For the purpose of consistency with the clinical analyses that we were previously reported with this tumor model [8], an aliquot of the exact same preparation of GL261 glioma cells (H-2^b^ haplotype) was used for inoculation into mice in this study as in the prior study. While these tumor cells had been engineered to express luciferase to enable clinical assessment of tumor development in the brain, the impact of luciferase was minimized in the experimental design by inoculating the same cell stock into mice that were to be either mock-vaccinated or receive the semi-allogenic vaccine. GL261 cells were maintained by passage in DMEM supplemented with 10% of fetal bovine serum. Generation of the RAGxGL261 semi-allogeneic hybrid vaccine was previously described [8]. Briefly, GL261 and RAG-neo cells (H-2^d^ haplotype), were mixed in serum-free medium containing 50 μM sodium dodecyl sulfate and centrifuged at 300× *g* for 5 min. The mixed cell pellet was then suspended in 50% polyethylene glycol (PEG)-1450 over a 1 min period while gently stirring. The cell suspension was then diluted over a 2 min period with DMEM supplemented with 10% FBS. After cell washing, cells were plated in selection medium containing 400 µg/mL G418 and HAT supplement (Invitrogen). Under these culture conditions only RAG×GL261 semi-allogeneic somatic cell hybrids can survive, since RAG-neo cells are killed by aminopterin and GL261 cells are killed by G418.

### 4.3. Experimental Protocol

Mouse studies were reviewed and approved by the Institutional Animal Care and Use Committee (IACUC) and were conducted in accordance with all applicable national and institutional guidelines for the care and use of animals. Ten to twelve week-old C57BL/6 male mice (Jackson Laboratories, Bar Harbor, ME, USA) were anesthetized by i.p. (intraperitoneal) injection of 90 mg/kg ketamine (JHP Pharmaceuticals, Rochester, MI, USA) and 10 mg/kg xylazine (Lloyd Laboratories, Shenandoah, IA, USA). Mice were then positioned in a Kopf stereotaxic frame for intracranial injection into the striatum (2.2 mm medio-lateral, 0.2 mm anterior-posterior, and 3 mm dorso-ventral). GL261 cells suspended in PBS (2 × 10^4^/mouse) were injection into the right cerebral hemisphere using a 5 μL Hamilton micro-syringe under mechanical control to avoid brain injuries during the procedure. Three days after tumor inoculation, treated mice were injected s.c. with lethally irradiated (30 Gray) GL261×RAG-neo hybrids (10^6^ cells per mouse) in 0.5 mL PBS, and mock-vaccinated mice were injected s.c. with 0.5 mL phosphate-buffered saline alone. Mice were checked daily until the end of the experiment. As mock-vaccinated mice exhibited signs of tumor burden, they were euthanized along with control mice and vaccinated tumor-bearing mice. Thus, each time the study was conducted, the time between tumor inoculation and when mice were sacrificed was the same for both the mock-vaccinated and the tumor-vaccinated mice. At time of sacrifice, spleens from mice in each group were collected and used as described below. Spleen cells production of cytokines was measured for each mouse individually. In total, spleen cell cultures were established for cytokine assessment from 6 control mice, 4 mock-vaccinated glioma-bearing mice, and 13 glioma-bearing mice that received the semi-allogeneic vaccine. This was conducted over the course of 4 separate experiments.

### 4.4. Spleen Processing

Spleens were collected at sacrifice from three groups of mice: (1) control mice; (2) GL261-derived tumor-bearing mice mock-vaccinated with phosphate-buffered saline; and (3) GL261-derived tumor-bearing mice vaccinated by subcutaneous (s.c.) injection of irradiated GL261×RAG-neo semi-allogeneic hybrids (10^6^ cells per mouse) in PBS [8]. Spleen cell suspensions were prepared using a Dounce homogenizer. Cells were passed through a 70 μm cell strainer (BD Biosciences, San Jose, CA, USA) and centrifuged at 4 °C for 5 min at 300× *g*. The cell pellet was suspended in 2 mL of red blood cells lysing buffer (ammonium-chloride-potassium (ACK), LONZA, Allendale, NJ, USA) for 3 min and diluted with 50 mL Hank’s balanced salt solution (HBSS) to inactivate ACK. The cell suspension freed of red blood cells was used for in vitro studies.

### 4.5. Analysis of Spleen Cell Cytokine Production

Spleen cells from each of the groups of mice were cultured in 1 mL in 24-well anti-CD3-coated tissue culture plates at 1 × 10^6^ cells/well in fresh medium (unstimulated) or in a 1:2 dilution of medium conditioned for 24 h by subconfluent cultures of GL261 tumor cells (stimulated). After 3 days of culture, supernatants were collected from the spleen cells and used to measure production of cytokines. Levels of cytokines in GL261-conditioned medium were also measured to control for cytokine levels that may have been contributed by the tumor cells. Levels of cytokines in the culture supernatants were measured using mouse cytometric bead array flex sets according to the instructions of the manufacturer (BD Biosciences, San Jose, CA, USA). A FACS Canto (BD Biosciences) flow cytometer was used to quantify cytokine profiles and relative amounts of each cytokine were determined using FCAP Array Software (manufactured by Soft Flow, Inc., St. Louis Park, MN, USA for BD Biosciences).

### 4.6. Statistical Analysis

Data were reported as means ± standard error of the mean. The Mann-Whitney *U* test was used to determine significance of differences in values between each of two parameters (GraphPad Prism version 6.03 for Windows, GraphPad Software, La Jolla, CA, USA). Significance was reported in the 95% confidence interval. Due to the differences in the number of animals in each group, the Hedges’ *g* test was used to determine the strength of the effect size as it provides a measure of effect size weighted according to the relative size of each sample.

## 5. Conclusions

The results of these experimental studies show that, for most of the cytokines measured, tumor-conditioned medium stimulated an overall increase in cytokine production by control spleen cells and spleen cells from vaccinated mice. In contrast, spleen cells of tumor-bearing, mock-vaccinated mice produced diminished levels of cytokines in the presence of tumor-conditioned medium. Thus, these results show that the vaccination resulted in a restoration of spleen cell cytokine production in response to mediators produced by tumor cells, as seen by a restored capacity to produce a broad spectrum of cytokines in response to the presence of tumor-derived products.
